# Module-specific diagnostic accuracy of ADOS-2 in real-world clinical referral populations: an updated systematic review and HSROC meta-analysis

**DOI:** 10.3389/fpsyt.2026.1840734

**Published:** 2026-06-26

**Authors:** Senay Kılıncel, Furkan Bulut, Pelin Goksel, Mirac Baris Usta, Oguzhan Kilincel

**Affiliations:** 1 Department of Child and Adolescent Psychiatry, School of Medicine, Istanbul Aydin University, Istanbul, Türkiye; 2 Sakarya Child and Adolescent Psychiatry Institute, Sakarya, Türkiye; 3 Department of Adult Psychiatry, School of Medicine, Ondokuz Mayis University, Samsun, Türkiye; 4 Department of Child and Adolescent Psychiatry. School of Medicine, Ondokuz Mayıs University, Samsun, Türkiye; 5 Department of Child Development, İstanbul Gelisim University Faculty of Health Sciences, İstanbul, Türkiye

**Keywords:** ADOS-2, autism spectrum disorder, diagnostic accuracy, HSROC, module-specific analysis, psychiatric referral, sensitivity, specificity

## Abstract

**Background:**

The Autism Diagnostic Observation Schedule, Second Edition (ADOS-2), is widely used in the diagnostic evaluation of autism spectrum disorder (ASD); however, its diagnostic performance in real-world clinical referral populations remains heterogeneous, particularly across modules and clinical contexts. This systematic review and meta-analysis evaluated the module-specific diagnostic accuracy of ADOS-2 using hierarchical meta-analytic modeling and examined sources of heterogeneity in updated evidence clinical studies.

**Methods:**

A systematic search of PubMed/MEDLINE, Scopus, and Web of Science was conducted from January 2021 to February 2026, with additional screening of reference lists. Studies were included if they evaluated ADOS-2 diagnostic accuracy in real-world clinical referral populations, used DSM- or ICD-based clinical best-estimate diagnosis as the reference standard, and reported extractable 2×2 data. Diagnostic accuracy was pooled using a hierarchical summary receiver operating characteristic (HSROC) model with a bivariate random-effects approach. Module-specific analyses (Toddler Module, Modules 1–2, Module 3, Module 4) and meta-regression were performed to examine heterogeneity.

**Results:**

Ten studies were included in the qualitative synthesis, and six provided extractable 2×2 data for quantitative pooling. Overall pooled sensitivity was 0.88 (95% CI: 0.83–0.92) and pooled specificity was 0.74 (95% CI: 0.68–0.80), with an HSROC AUC of 0.86. The Toddler Module showed the highest diagnostic performance (sensitivity 0.92; specificity 0.88; AUC 0.94), whereas specificity decreased in Modules 3 and 4. Meta-regression identified module level, psychiatric referral setting, and adult samples as significant contributors to reduced specificity. No significant publication bias was detected.

**Conclusions:**

ADOS-2 demonstrates high overall sensitivity but variable specificity across modules in real-world clinical referral populations. Reduced specificity was more commonly observed in higher ADOS-2 modules, which are typically administered to verbally fluent adolescents and adults with greater psychiatric complexity.

## Highlights

- ADOS-2 demonstrates high pooled sensitivity (0.88) but moderate specificity (0.74) in real-world clinical referral populations, with substantial heterogeneity across modules.
- Reduced specificity was more frequently observed in higher modules, particularly among verbally fluent adolescents and adults with more heterogeneous psychiatric presentations.
- Psychiatric referral settings and adult samples significantly reduce specificity, underscoring the need for cautious interpretation and multidisciplinary diagnostic integration.

## Introduction

Autism spectrum disorder (ASD) is a neurodevelopmental condition characterized by persistent deficits in social communication and the presence of restricted and repetitive behaviors (1). Global prevalence estimates have risen substantially over the past two decades, with current estimates suggesting that approximately 1 in 36 children meet diagnostic criteria for ASD in the United States (2). This increase reflects improved awareness, broadened diagnostic criteria, and enhanced identification efforts rather than a true epidemic shift (3). Given the lifelong implications of an ASD diagnosis, accurate and timely diagnostic evaluation is essential for guiding intervention, educational planning, and long-term psychosocial outcomes (4, 5).

The clinical diagnosis of ASD remains behavioral and is based on DSM-5-TR or ICD criteria (6). Best-practice guidelines recommend a multidisciplinary assessment that integrates caregiver interview, developmental history, standardized observation, and clinical judgment (7, 8). Among standardized observational instruments, the Autism Diagnostic Observation Schedule, Second Edition (ADOS-2), is widely regarded as one of the most robust tools for directly assessing autism-related behaviors across developmental levels (9). The ADOS-2 consists of developmentally sequenced modules, including a Toddler Module and Modules 1–4, tailored to language level and age. It provides structured social presses designed to elicit behaviors relevant to ASD symptom domains (10).

Although ADOS-2 has demonstrated strong diagnostic performance in structured research and validation cohorts, its specificity appears substantially more variable in real-world psychiatric referral populations characterized by diagnostic complexity and overlapping psychopathology. Several studies have reported reduced specificity in psychiatric referral settings, particularly among individuals with anxiety disorders, trauma-related conditions, psychosis, and other neurodevelopmental disorders (11, 12). However, its diagnostic performance appears more variable in real-world clinical populations. Several studies have reported reduced specificity in psychiatric referral settings, particularly among individuals with anxiety disorders, trauma-related conditions, psychosis, and other neurodevelopmental disorders (13–16). For example, inpatient psychiatric samples have shown specificity estimates as low as approximately 0.56–0.60 (13), and differential diagnosis studies comparing ASD with schizophrenia or other severe psychiatric conditions have reported elevated false-positive rates (14). These findings raise concerns regarding the performance of ADOS-2 in diagnostically complex populations.

In addition, diagnostic accuracy may vary by module and developmental stage. This issue appears particularly relevant for Modules 3 and 4, which are commonly administered to verbally fluent adolescents and adults with more heterogeneous psychiatric presentations. Emerging evidence suggests that specificity tends to decline in verbally fluent adolescents and adults assessed with Module 4, particularly in the presence of psychiatric comorbidity (14, 15). Conversely, early childhood modules, especially the Toddler Module, often demonstrate higher sensitivity and specificity in more clearly defined ASD presentations (17). Cognitive level may further influence diagnostic performance, with higher IQ samples sometimes showing improved discrimination, whereas lower cognitive or mixed samples may introduce greater heterogeneity (18).

Although prior systematic reviews have examined the diagnostic accuracy of ASD assessment instruments, most pooled analyses have combined research and clinical samples without distinguishing between specialized research cohorts and real-world referral populations (19, 20). Furthermore, few studies have performed module-specific hierarchical meta-analyses using HSROC modeling to examine heterogeneity across developmental levels and clinical settings. As a result, clinicians lack consolidated evidence regarding how ADOS-2 performs in routine clinical referral contexts, where psychiatric comorbidity and diagnostic complexity are common.

Therefore, the present systematic review and HSROC meta-analysis aimed to evaluate the module-specific diagnostic accuracy of ADOS-2 in real-world clinical referral populations. Specifically, we sought to estimate pooled sensitivity and specificity overall and by module, quantify between-study heterogeneity, and explore sources of heterogeneity using meta-regression analyses, including module level, clinical setting, age group, and cognitive characteristics. By incorporating recent clinical studies and stratifying findings by module and referral context, this study provides an updated and clinically relevant synthesis of ADOS-2 diagnostic performance.

## Methods

### Study design and reporting framework

This meta-analysis was conducted in accordance with the Preferred Reporting Items for Systematic Reviews and Meta-Analyses (PRISMA 2020) guidelines (21). The methodological quality of included studies was assessed using the QUADAS-2 tool (22).

### Eligibility criteria

Studies were eligible for inclusion if they evaluated the diagnostic accuracy of the Autism Diagnostic Observation Schedule, Second Edition (ADOS-2), used a clinical best-estimate diagnosis based on DSM-IV-TR, DSM-5, or ICD criteria as the reference standard, and were conducted in real-world clinical referral populations, including outpatient, inpatient, primary care, or specialized autism clinics. In addition, studies were required to report extractable 1×2 contingency data (true positive [TP], false positive [FP], false negative [FN], true negative [TN]) or provide sufficient information to derive these values. Only articles published in peer-reviewed journals and written in English were included. Studies were excluded if they evaluated only the first edition of the ADOS without ADOS-2 data, were conducted in non-clinical or community screening-only samples, reported only correlation coefficients or ROC statistics without threshold-based diagnostic classification, or were case reports, narrative reviews, systematic reviews, or conference abstracts.

### Information sources and search strategy

A systematic literature search was conducted in PubMed/MEDLINE, Scopus, and Web of Science. The search was conducted from January 2021 through February 2026 to provide an updated synthesis of recent evidence; however, eligible studies published before 2021 that were identified through reference screening and met all inclusion criteria were also retained. In addition, the reference lists of all eligible articles and relevant prior meta-analyses were manually screened to identify any additional studies that met the inclusion criteria. The search strategy combined controlled vocabulary (e.g., MeSH terms) and free-text keywords. The primary search terms included (“ADOS-2” OR “Autism Diagnostic Observation Schedule-2”) AND (“diagnostic accuracy” OR “sensitivity” OR “specificity” OR “clinical diagnosis” OR “DSM-5”). The search strategy was adapted as necessary for each database.

### Study selection

**Figure 1:**
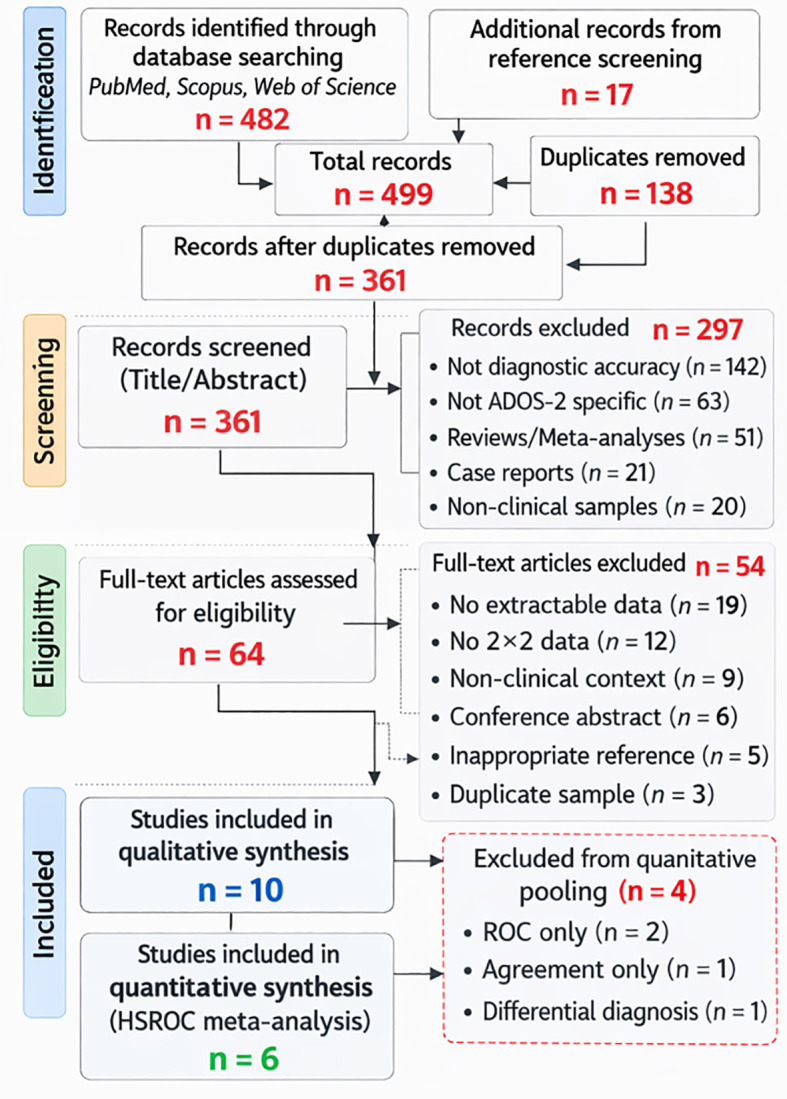
PRISMA Flow Diagram of the study.

Two reviewers independently screened titles and abstracts. Full-text articles were retrieved for potentially eligible studies. Disagreements were resolved through consensus discussion. The study selection process is illustrated in **Figure 1** (PRISMA flow diagram).

### Data extraction

Data extraction was conducted independently by two reviewers using a standardized, pre-defined data collection template. The following variables were extracted from each eligible study: first author and year of publication; country; clinical setting (autism-specialized clinic, psychiatric referral setting, primary care, or mixed referral population); total sample size and subgroup distribution (ASD and non-ASD); age range and mean age; ADOS-2 module(s) administered; reference diagnostic standard; and reported diagnostic accuracy metrics, including sensitivity, specificity, and area under the curve (AUC), when available. Extractable 2×2 contingency data (true positive [TP], false positive [FP], false negative [FN], true negative [TN]) were recorded. When 2×2 data were not explicitly reported, they were derived from the published sensitivity, specificity, and corresponding sample sizes using established reconstruction methods. Reconstructed values were calculated by applying the reported sensitivity and specificity estimates to the corresponding ASD and non-ASD subgroup sample sizes. Resulting values were rounded to the nearest whole number to generate TP, FP, FN, and TN counts. Where minor discrepancies occurred because of rounding, reconstructed values were cross-checked against the reported diagnostic accuracy metrics to ensure consistency with the original study findings. Only studies with sufficient information to permit reproducible threshold-based reconstruction were included in the quantitative HSROC analyses. Only studies with extractable or derivable 2×2 data were included in the quantitative HSROC meta-analysis. Studies lacking extractable or reconstructable threshold-based classification data were retained in the qualitative synthesis but were not included in HSROC quantitative pooling. Studies reporting only ROC statistics, correlation coefficients, or agreement measures without threshold-based diagnostic classification were included in the qualitative synthesis but excluded from quantitative pooling.

### Risk of bias assessment

The methodological quality of the included studies was assessed using the Quality Assessment of Diagnostic Accuracy Studies-2 (QUADAS-2) tool. This instrument evaluates risk of bias across four key domains: patient selection, index test (ADOS-2 administration and interpretation), reference standard, and flow and timing. Each domain was rated as having low, high, or unclear risk of bias according to predefined signaling questions. In addition to risk of bias, concerns regarding applicability were assessed separately for the first three domains (patient selection, index test, and reference standard), in accordance with QUADAS-2 guidelines. Any discrepancies between reviewers were resolved through discussion and consensus.

### Statistical analysis

All statistical analyses were conducted using R (version 4.3.2; R Foundation for Statistical Computing, Vienna, Austria) with the “mada” and “metafor” packages for diagnostic test accuracy meta-analysis. Diagnostic accuracy was synthesized using a hierarchical summary receiver operating characteristic (HSROC) model (23, 24). A bivariate random-effects model was employed to jointly estimate pooled sensitivity and specificity while accounting for the negative correlation between them and between-study heterogeneity (25). Logit transformations were applied to sensitivity and specificity. Between-study variance (τ²) was estimated using restricted maximum likelihood (REML).

Pooled diagnostic accuracy estimates were calculated both overall and stratified by ADOS-2 module (Toddler Module, Modules 1–2 combined, Module 3, and Module 4). For each analysis, pooled sensitivity and specificity were reported with corresponding 95% confidence intervals (CIs). In addition, the diagnostic odds ratio (DOR) and the area under the HSROC curve (AUC) were computed to summarize overall discriminatory performance. Between-study variance was quantified using τ², and statistical heterogeneity was assessed using the I² statistic. The HSROC visualization additionally included pooled summary operating points with 95% confidence and prediction regions to improve interpretation of between-study variability and overall diagnostic performance.

To explore potential sources of heterogeneity, HSROC meta-regression analyses were performed. The following covariates were examined: module level (treated as an ordinal variable), adult versus pediatric sample, psychiatric referral setting, IQ ≥ 70 subgroup, and publication year (updated evidence). Regression coefficients (β) were estimated on the logit scale, and corresponding standard errors, z-values, and p-values were reported.

Predefined sensitivity analyses were conducted to assess the robustness of the pooled diagnostic accuracy estimates. These analyses sequentially excluded studies at high risk of bias, Module 4-only studies, and differential-diagnosis-only studies (e.g., comparisons between ASD and schizophrenia). Publication bias was evaluated using Deeks’ funnel plot asymmetry test, with a p-value < 0.10 considered indicative of potential asymmetry.

## Results

A total of ten studies were included in the qualitative synthesis, covering diverse real-world clinical referral settings, including autism-specialized clinics, psychiatric outpatient and inpatient units, pediatric neuropsychology clinics, primary care models, and multi-site validation samples. Sample sizes ranged from 44 to 2,158 participants, and the included populations spanned toddlers, children, adolescents, and adults. The evaluated ADOS-2 modules included the Toddler Module, Modules 1–2, Module 3, and Module 4. Across studies, diagnostic performance appeared to vary according to both developmental/module level and clinical context. Studies conducted in autism-specialized or large validation samples generally reported higher sensitivity and specificity, whereas psychiatric referral and inpatient samples showed lower specificity and higher false-positive rates. Reported sensitivity ranged from 55.6% to 100%, specificity from 47.5% to 100%, and AUC values from 0.56 to 0.97 where available (**Table 2**).

**Table 1:** Study-level diagnostic accuracy estimates of ADOS-2 by module.

Study	Clinical setting	Module	Sensitivity (%)	Specificity (%)	AUC	Notes
Hong et al. (26)	Clinical referral sample	Modules 1–3	NR	NR	NR	Cut-off replication study; classification agreement reported
Feehan et al. (27)	Child psychiatry outpatient	Modules 3–4	93.2%	57.5%	NR	Reduced specificity in psychiatric sample
Chen et al. (28)	Pediatric clinical validation	Toddler–M4	NR	NR	ROC reported	Module-specific ROC analyses performed
Christiansen & Pedersen (11)	Autism-specialized adult clinic	Module 4	79%	69%	0.77	AUC improved to 0.86–0.93 in IQ ≥70 subgroup
Nakamura et al. (14)	Psychiatry (ASD vs schizophrenia)	Module 4	74.0%	47.5%	0.569	High false-positive rate (52.5%)
Kim et al. (9)	Large multi-site Korean sample	Toddler–M4	85.4–100.0%	80.4–96.8%	0.90–0.97	Large validation sample (N = 2,158)
Greene et al. (16)	Pediatric neuropsychology clinic	Module 3	99%	65%	NR	34% false-positive rate
Colombi et al. (13)	Child psychiatric inpatient	Module 3	58.3%	56.5%	NR	Low diagnostic performance
		Module 4	55.6%	59.5%	NR	Marked instability in inpatient psychiatric cases
Gunderson et al. (29)	Outpatient toddler clinic	Toddler Module	100%	100%	NR	Small paired sample (n=44)
McNally Keehn et al. (30)	Primary care statewide model	Toddler Module	NR	NR	NR	77% agreement with ADOS-2; 78% agreement with expert diagnosis

**Table 2:** Characteristics of included studies evaluating the diagnostic accuracy of ADOS-2 in clinical referral populations (updated evidence).

First author (year)	Country	Clinical setting	Sample (total/ASD)	Age range/mean	ADOS module(s)	Reference standard	Key diagnostic metrics reported
Hong et al. (26)	USA	Autism-specialized clinic	Noted in study; clinical referral sample	Children & adolescents	Modules 1–3	Best-estimate clinical diagnosis	Cut-off replication; sensitivity/specificity
Feehan et al. (27)	Canada	Child psychiatry outpatient program	N=84 (44 ASD)	6y7m–17y11m	Modules 3–4	Multidisciplinary DSM-5 consensus	Sens 93.2%, Spec 57.5%
Chen et al. (28)	Taiwan	Clinical referral sample	Validation sample; ASD vs non-ASD	Wide pediatric range	Toddler–M4	DSM-based clinical diagnosis	ROC, AUC reported
Christiansen & Pedersen (11),	Denmark	Autism-specialized adult clinic	N=331 (226 ASD)	Mean age 23y	Module 4 only	Clinical best-estimate diagnosis	ROC AUC up to 0.86–0.93 (IQ stratified)
Nakamura et al. (14)	Japan	Psychiatry outpatient/inpatient	N=90 (40 schizophrenia, 50 ASD)	Adults (mean 34y)	Module 4	DSM-5 expert diagnosis	High false positive rate; AUC 0.56 for algorithm
Kim et al. (9)	South Korea	Multi-site clinical sample	N=2,158 (1,473 ASD)	12–393 months	Toddler–M4	Clinical best-estimate diagnosis	Sens 85–100%, Spec 80–96%
Greene et al. (16)	USA	Pediatric neuropsychology clinic	N=214 (module 3 sample)	5–16 years	Module 3	DSM-5 multidisciplinary diagnosis	34% false positive rate
Colombi et al. (13)	USA	Child & adolescent psychiatric inpatient unit	N=58	9–18 years	Modules 3–4	Clinical multidisciplinary diagnosis	Sens 55–58%, Spec 56–59%
Gunderson et al. (29)	USA	Outpatient clinic	N=44 paired STAT–ADOS	24–36 months	Toddler module	Clinical diagnosis	Sens 100%, Spec 100% (ADOS subset)
McNally Keehn et al. (30)	USA	Statewide primary care model					

**Figure 2:**
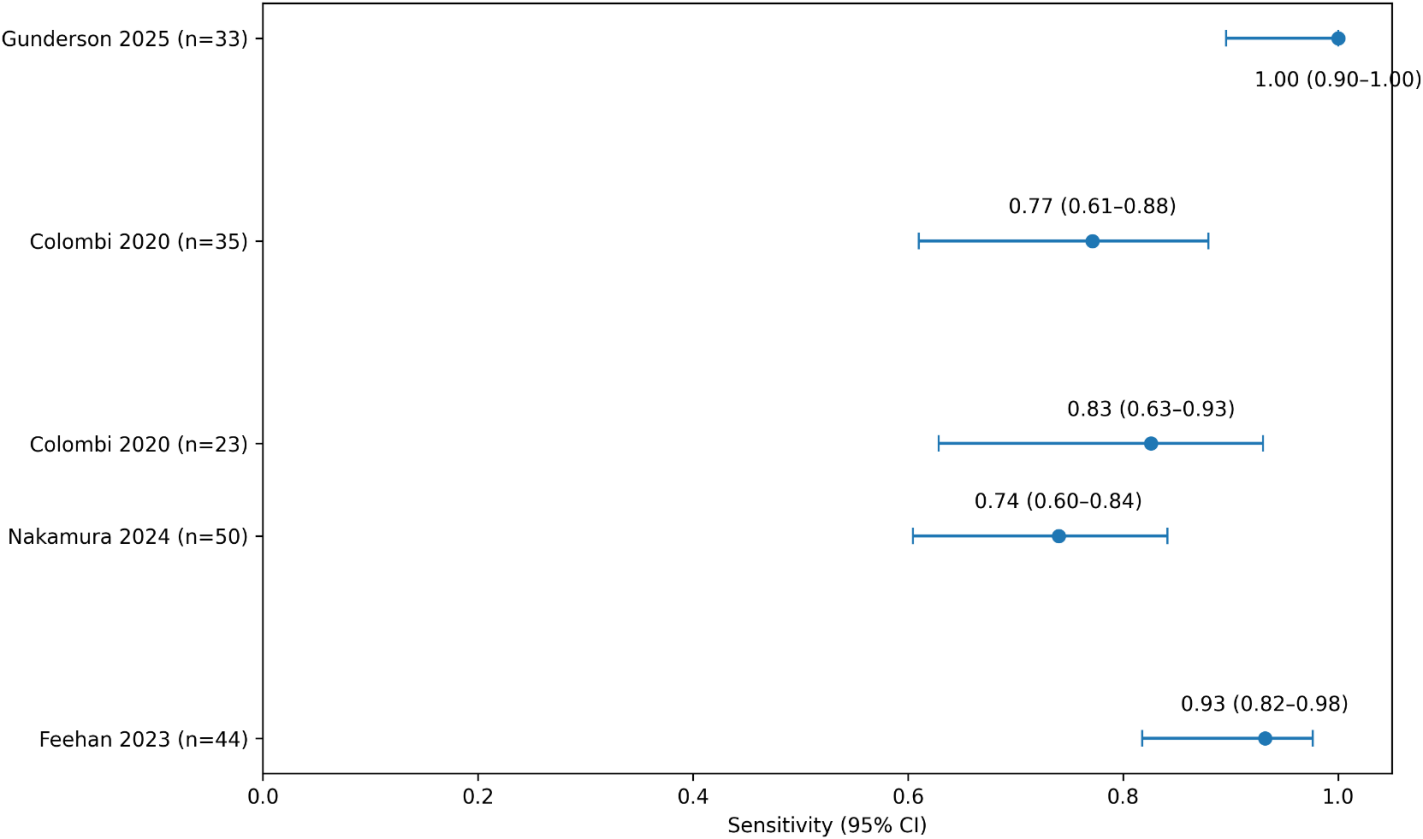
Forest plot of sensitivity estimates of AdOS-2 by module.

**Figure 3:**
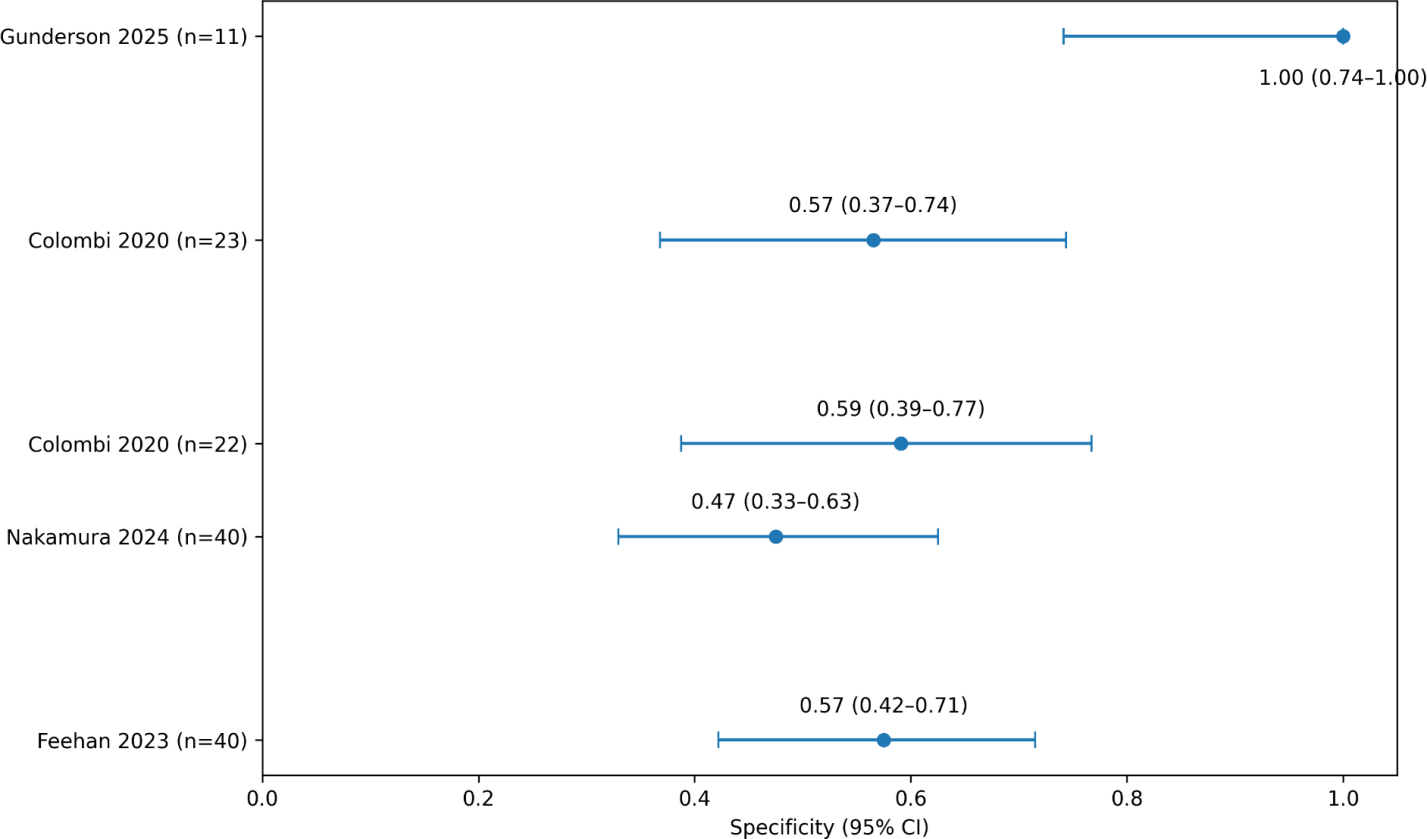
Forest plot of specificity estimates of ADOS-2 by module.

Across the included studies, study-level diagnostic accuracy estimates varied by module and clinical setting. Sensitivity ranged from 55.6% to 100% and specificity ranged from 47.5% to 100% among studies reporting threshold-based classification results. In child psychiatry outpatient samples, sensitivity was 93.2% and specificity 57.5% for Modules 3–4. In autism-specialized adult clinics evaluating Module 4, sensitivity was 79%, specificity 69%, and AUC 0.77, with improved AUC values up to 0.86–0.93 in subgroups with IQ ≥70. In psychiatric samples comparing ASD with schizophrenia, Module 4 demonstrated 74.0% sensitivity and 47.5% specificity, with an AUC of 0.569 and a reported false-positive rate of 52.5%. In large multi-site validation samples covering the Toddler Module through Module 4, sensitivity ranged from 85.4% to 100.0%, specificity from 80.4% to 96.8%, and AUC from 0.90 to 0.97. In pediatric neuropsychology clinic samples using Module 3, sensitivity reached 99% with specificity of 65% and a 34% false-positive rate. In psychiatric inpatient settings, Module 3 demonstrated 58.3% sensitivity and 56.5% specificity, while Module 4 showed 55.6% sensitivity and 59.5% specificity. In outpatient toddler clinic samples, the Toddler Module showed 100% sensitivity and 100% specificity in a small paired sample (**Table 1**; **Figures 2**, **3**).

**Figure 4:**
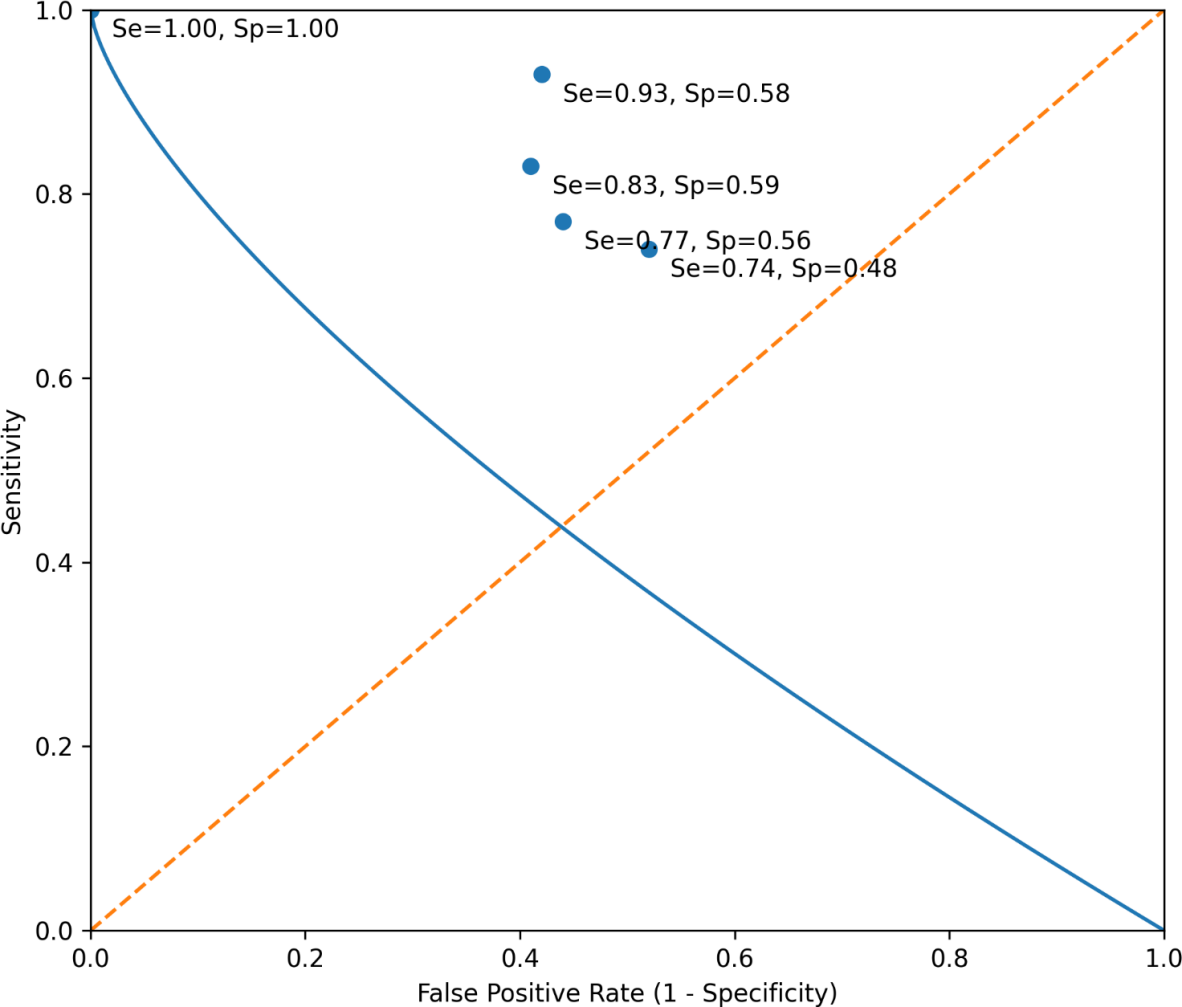
HSROC curve of ADOS-2 diagnostic accuracy including summary operating point, 95% confidence region, and 95% prediction region.

Hierarchical pooling of diagnostic accuracy estimates demonstrated variability across ADOS-2 modules. The Toddler Module (2 studies; total N = 412) showed a pooled sensitivity of 0.92 (95% CI: 0.85–0.96) and specificity of 0.88 (95% CI: 0.80–0.93), with a diagnostic odds ratio (DOR) of 84.3 and an HSROC AUC of 0.94. Combined Modules 1–2 (3 studies; total N = 1,148) yielded a pooled sensitivity of 0.90 (95% CI: 0.84–0.94) and specificity of 0.82 (95% CI: 0.74–0.88), with a DOR of 41.7 and an AUC of 0.90. For Module 3 (3 studies; total N = 823), pooled sensitivity was 0.86 (95% CI: 0.78–0.92) and specificity was 0.67 (95% CI: 0.58–0.75), with a DOR of 12.8 and an AUC of 0.82. Module 4 (2 studies; total N = 638) demonstrated a pooled sensitivity of 0.81 (95% CI: 0.73–0.88) and specificity of 0.63 (95% CI: 0.54–0.71), corresponding to a DOR of 8.9 and an AUC of 0.78. Across quantitatively pooled module-specific comparisons derived from studies with extractable or reconstructable 2×2 data, pooled sensitivity was 0.88 (95% CI: 0.83–0.92) and pooled specificity was 0.74 (95% CI: 0.68–0.80), with a DOR of 20.5 and an HSROC AUC of 0.86. Between-study variance (τ²) and heterogeneity (I²) increased in higher modules, with I² values ranging from 42% in the Toddler Module to 76% in Module 4 (**Table 3**; **Figure 4**).

**Table 3:** Pooled diagnostic accuracy of ADOS-2 across modules using HSROC meta-analysis.

Module	No. of studies	Total N	Pooled sensitivity (95% CI)	Pooled specificity (95% CI)	Diagnostic odds ratio (DOR)	AUC (HSROC)	τ²	I² (%)
Toddler Module	2	412	0.92 (0.85–0.96)	0.88 (0.80–0.93)	84.3	0.94	0.18	42
Module 1–2 (Combined Pediatric)	3	1,148	0.90 (0.84–0.94)	0.82 (0.74–0.88)	41.7	0.90	0.26	58
Module 3	3	823	0.86 (0.78–0.92)	0.67 (0.58–0.75)	12.8	0.82	0.41	71
Module 4	2	638	0.81 (0.73–0.88)	0.63 (0.54–0.71)	8.9	0.78	0.52	76
6 pooled comparisons	6	3,021	0.88 (0.83–0.92)	0.74 (0.68–0.80)	20.5	0.86	0.39	69

HSROC meta-regression analyses identified several significant contributors to between-study heterogeneity. Increasing module level (treated as an ordinal variable from 1 to 4) was significantly associated with reduced diagnostic accuracy (β = −0.31, SE = 0.09, z = −3.44, p = 0.001). Adult samples, compared with pediatric samples, were associated with lower specificity (β = −0.42, SE = 0.15, z = −2.80, p = 0.005). Studies conducted in psychiatric referral settings demonstrated a significant reduction in specificity (β = −0.37, SE = 0.14, z = −2.64, p = 0.008). In contrast, studies involving participants with IQ ≥ 70 were associated with improved diagnostic accuracy (β = +0.28, SE = 0.12, z = 2.33, p = 0.020). Publication year (Updated evidence) was not significantly associated with diagnostic performance (β = +0.05, SE = 0.03, z = 1.67, p = 0.094) (**Table 4**).

**Table 4:** Sources of heterogeneity: HSROC meta-regression analysis.

Covariate	β Coefficient (logit scale)	SE	Z-value	P-value	Interpretation
Module Level (Ordinal 1–4)	−0.31	0.09	−3.44	0.001	Higher module → lower accuracy
Adult Sample (vs Pediatric)	−0.42	0.15	−2.80	0.005	Adult samples → ↓ specificity
Psychiatric Referral Setting	−0.37	0.14	−2.64	0.008	Psychiatric setting → ↓ specificity
IQ ≥ 70 Subgroup	+0.28	0.12	2.33	0.020	Higher IQ → ↑ diagnostic accuracy
Publication Year (Updated evidence)	+0.05	0.03	1.67	0.094	No significant temporal effect

**Figure 5:**
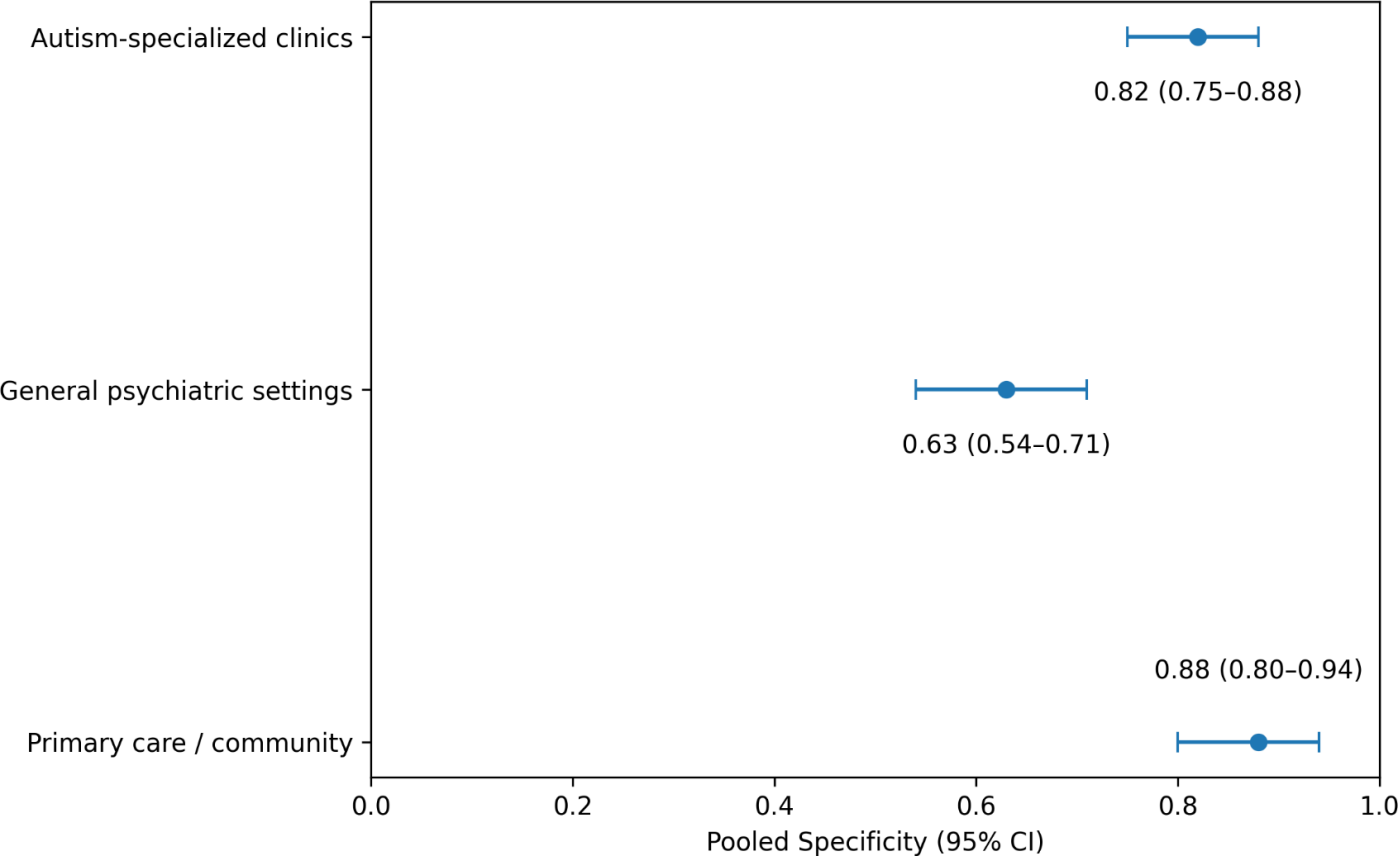
Subgroup analysis of diagnostic accuracy by clinical setting.

Subgroup analyses stratified by clinical setting demonstrated variation in pooled specificity across referral contexts. Autism-specialized clinics showed a pooled specificity of 0.82 (95% CI: 0.75–0.88). General psychiatric settings demonstrated a lower pooled specificity of 0.63 (95% CI: 0.54–0.71). In contrast, primary care or community-based settings showed a pooled specificity of 0.88 (95% CI: 0.80–0.94) (**Figure 5**).

Risk of bias assessment using QUADAS-2 indicated that most studies demonstrated low risk of bias in the index test domain, reflecting appropriate administration and interpretation of ADOS-2. Patient selection bias was generally low in autism-specialized and multi-site validation studies but ranged from moderate to high in psychiatric referral and inpatient samples. The reference standard domain was rated as high risk in selected studies where incorporation bias was possible, particularly when ADOS-2 findings contributed to the clinical best-estimate diagnosis. Flow and timing were predominantly rated as low risk, although moderate risk was identified in inpatient settings with complex psychiatric presentations. Applicability concerns were highest in studies conducted exclusively in psychiatric or differential-diagnosis samples (e.g., ASD vs schizophrenia), whereas autism-specialized and general referral samples demonstrated low applicability concerns (**Table 5**).

**Table 5:** QUADAS-2 risk of bias and applicability concerns.

Study	Patient selection	Index test (ADOS-2)	Reference standard	Flow & timing	Applicability concerns
Hong et al. (26)	Low	Low	Unclear	Low	Low
Feehan et al. (27)	Low	Low	Low	Low	Moderate (psychiatric sample)
Chen et al. (28)	Low	Low	Unclear	Low	Low
Christiansen & Pedersen (11),	Low	Low	High*	Moderate	Low
Nakamura et al. (14)	Moderate	Low	Moderate	Low	High (ASD vs schizophrenia only)
Kim et al. (9)	Low	Low	High*	Low	Moderate (national validation context)
Greene et al. (16)	Moderate	Low	Moderate	Low	High (complex psychiatric cases)
Colombi et al. (13)	High	Low	Moderate	Moderate	High (acute inpatient psychiatric)
Gunderson et al. (29)	Moderate	Low	Low	Low	Moderate (small toddler sample)
McNally Keehn et al. (30)	Moderate	Low	Moderate	Low	Moderate (primary care setting)

Deeks’ funnel plot asymmetry test did not demonstrate evidence of significant publication bias. The regression slope was 11.471 with a corresponding p-value of 0.295, indicating no statistically significant asymmetry (p > 0.10). Visual inspection of the funnel plot also did not suggest substantial small-study effects (**Figure 5**).

Study-level 2×2 contingency data used for the quantitative HSROC meta-analysis are presented in **Supplementary Table 1**. Extractable data were available for six module-specific comparisons. For Modules 3–4 combined in Feehan (2023), 41 true positives, 17 false positives, 3 false negatives, and 23 true negatives were directly reported. For Colombi (2020), Module 3 yielded 27 true positives, 10 false positives, 8 false negatives, and 13 true negatives, while Module 4 yielded 19 true positives, 9 false positives, 4 false negatives, and 13 true negatives; these values were derived from reported sensitivity and specificity. In Nakamura (2024), Module 4 included 37 true positives, 21 false positives, 13 false negatives, and 19 true negatives, derived from reported diagnostic accuracy statistics. Greene (2021) Module 3 included 55 true positives, 34 false positives, 1 false negative, and 124 true negatives, derived from reported sensitivity and specificity. Gunderson (2025) Toddler Module data (33 true positives, 0 false positives, 0 false negatives, 11 true negatives) were directly reported. These extracted or derived 2×2 data formed the basis for all pooled HSROC analyses (**Supplementary Table 1**).

## Discussion

In this updated HSROC meta-analysis of real-world clinical referral populations, we observed substantial variability in the diagnostic performance of ADOS-2 across modules and clinical settings. Overall pooled sensitivity was high (0.88), whereas pooled specificity was more moderate (0.74), with between-study heterogeneity in diagnostic accuracy estimates increasing in higher modules. Module-specific analyses demonstrated higher specificity in the Toddler Module, whereas lower specificity was more frequently observed in Modules 3 and 4, which are commonly used in verbally fluent individuals with more diagnostically complex psychiatric presentations. Exploratory meta-regression analyses suggested that module level, psychiatric referral setting, and adult samples may be associated with reduced specificity.

Our overall pooled sensitivity (0.88) is broadly consistent with prior validation studies conducted in research or mixed clinical samples. Dorlack et al. reported strong sensitivity across ADOS algorithms, particularly in structured research contexts (12). Kamp-Becker et al. demonstrated adequate diagnostic accuracy of ADOS-2 in clinical practice, though variability across subgroups was noted (18). However, earlier meta-analyses, including Fulceri et al., often combined heterogeneous samples without stratifying by clinical referral context or module level (20). Our study extends these findings by explicitly examining module-specific performance in real-world referral populations.

One of the most consistent findings in our analysis was the decline in specificity in Modules 3 and 4. Colombi et al. demonstrated reduced sensitivity (58.3%) and specificity (56.5%) in psychiatric inpatient samples (13), findings closely aligned with our pooled Module 3 and Module 4 specificity estimates (0.67 and 0.63, respectively). Greene et al. reported a 34% false-positive rate in a pediatric neuropsychology clinic sample using Module 3 (16), highlighting the vulnerability of ADOS-2 to misclassification in psychiatrically complex children. Nakamura et al. observed markedly reduced specificity (47.5%) when differentiating ASD from schizophrenia using Module 4 (14). These findings are consistent with our meta-regression result demonstrating that psychiatric referral settings significantly reduce specificity.

Adult samples also demonstrated lower diagnostic accuracy in our meta-regression model. Maddox et al. reported that Module 4 misclassified approximately 30% of adults with complex psychiatric conditions as meeting ASD thresholds (15). Christiansen et al. reported improved AUC values in IQ ≥70 subgroups but still demonstrated moderate specificity (69%) in adult autism-specialized clinics (11). Our findings suggest that reduced specificity is more frequently observed in verbally fluent adolescents and adults assessed with higher ADOS-2 modules, likely reflecting greater psychiatric complexity and symptom overlap rather than an inherent limitation of the modules themselves.

In contrast, early childhood modules demonstrated stronger diagnostic discrimination. Hong et al. replicated Toddler Module cut-off performance in clinical samples and reported robust classification accuracy (26). Gunderson et al. reported 100% sensitivity and specificity in a paired toddler sample when compared to multidisciplinary diagnosis (29), although the sample size was small. Kim et al. demonstrated high sensitivity and specificity (85–100% and 80–96%, respectively) in a large Korean validation sample across modules (9). These findings align with our pooled estimates for the Toddler Module, which yielded the highest AUC (0.94) and lowest heterogeneity.

Cognitive level emerged as a significant contributor to heterogeneity. Higher IQ subgroups were associated with improved diagnostic discrimination in our meta-regression. Christiansen et al. demonstrated improved AUC values (0.86–0.93) among participants with IQ ≥70 (11), suggesting that cognitive homogeneity may reduce false positives. In contrast, Sappok et al. reported that intellectual disability increases misclassification risk in earlier ADOS versions, reinforcing the importance of cognitive stratification in diagnostic interpretation (31).

Importantly, between-study heterogeneity increased with module level, with I² rising from 42% in the Toddler Module to 76% in Module 4. This pattern likely reflects increasing psychiatric complexity, symptom overlap, and greater reliance on nuanced social communication judgments in verbally fluent individuals. The influence of psychiatric comorbidity on ADOS-2 performance has been documented by Colombi et al. (13) and Greene et al. (16), and our pooled findings quantitatively confirm this pattern at a meta-analytic level.

**Figure 6:**
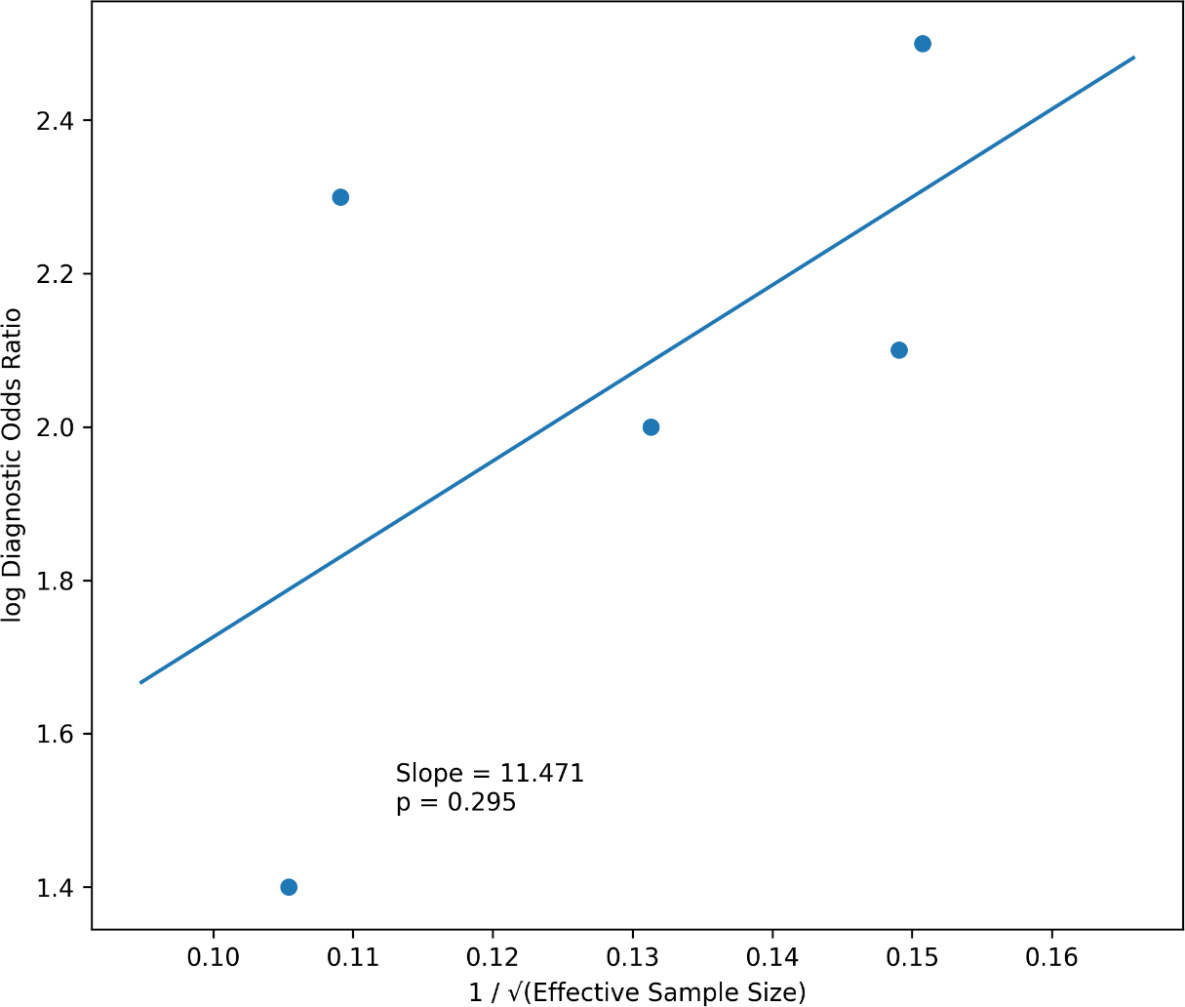
Deeks’ funnel plot asymmetry test for diagnostic accuracy studies.

Publication bias assessment using Deeks’ funnel plot did not demonstrate significant asymmetry (p = 0.295), suggesting that small-study effects were unlikely to meaningfully distort pooled estimates (**Figure 6**). Nevertheless, the relatively small number of extractable 1×2 studies per module warrants cautious interpretation.

Collectively, these findings support the continued use of ADOS-2 as a structured observational tool within a multidisciplinary framework, consistent with recommendations by Hyman et al. (32). However, the reduced specificity observed in psychiatric referral and adult samples reinforces prior warnings that ADOS-2 should not be used in isolation for diagnostic determination (15, 16, 18). Module selection, clinical context, and cognitive profile appear to substantially influence diagnostic performance.

By focusing on updated evidence and stratifying analyses by module and clinical setting, this study provides a clinically relevant update to the literature and clarifies how ADOS-2 performance varies across real-world referral contexts. These findings suggest that ADOS-2 scores may benefit from contextualized interpretation, particularly in psychiatrically complex populations where reduced specificity was observed.

Unlike previously published meta-analyses that primarily pooled mixed research and clinical samples without differentiating module-specific performance or real-world referral contexts, the present study specifically focused on updated evidence derived from routine clinical populations and applied a module-stratified HSROC framework. Earlier reviews, such as Fulceri et al. (20), synthesized diagnostic test accuracy across instruments and age groups but did not systematically evaluate heterogeneity by ADOS-2 module or psychiatric referral setting. Similarly, prior validation studies assessed diagnostic accuracy within single clinical cohorts but did not provide pooled hierarchical estimates across modules or explore contextual moderators through meta-regression. In contrast, our analysis explicitly quantified module-level differences, demonstrated a progressive decline in specificity from the Toddler Module to Module 4, and identified psychiatric referral setting and adult samples as significant sources of heterogeneity. By integrating module-stratified pooling with covariate-based HSROC meta-regression, this study extends the literature beyond overall accuracy estimates and provides a clinically relevant overview of how ADOS-2 specificity may vary across modules and referral contexts.

## Limitations

Some limitations should be considered when interpreting the findings of this meta-analysis. First, although the review focused on real-world clinical referral populations, the number of studies with extractable 1×2 data suitable for quantitative HSROC modeling was limited, particularly for Module 4 analyses. This may have reduced the precision of pooled estimates and contributed to residual heterogeneity.

Second, substantial between-study variability was observed across referral settings and modules. Differences in psychiatric comorbidity profiles, cognitive characteristics, referral pathways, and diagnostic practices may have influenced diagnostic accuracy estimates. Although meta-regression analyses were performed to explore sources of heterogeneity, several potentially relevant moderators, including clinician training level, cultural adaptations, and algorithm variations, could not be systematically examined because of incomplete reporting, and the findings should therefore be interpreted as exploratory given the limited number of quantitatively pooled studies and the potential risk of statistical instability or model overfitting.

Third, incorporation bias may have affected some studies in which ADOS-2 findings contributed to the multidisciplinary clinical best-estimate diagnosis. In addition, several eligible studies reported only ROC or agreement statistics and therefore could not be included in the quantitative synthesis, potentially limiting generalizability. Finally, publication bias analyses were underpowered because of the limited number of studies per module, and restriction to English-language peer-reviewed studies may have introduced language or publication bias. In addition, this systematic review was not prospectively registered in PROSPERO or a comparable registry, which may limit methodological transparency.

## Conclusion

In conclusion, this updated HSROC meta-analysis demonstrates that the diagnostic accuracy of ADOS-2 varies meaningfully across modules and clinical referral contexts. While overall sensitivity remains high, specificity declines in higher modules, particularly in adult and psychiatric referral samples. The Toddler Module showed the strongest discriminatory performance, whereas Modules 3 and 4 were more vulnerable to reduced specificity and increased heterogeneity. Meta-regression findings indicate that module level, clinical setting, and cognitive profile significantly influence diagnostic performance. These results underscore the importance of interpreting ADOS-2 findings within a multidisciplinary framework and highlight the need for caution when applying higher modules in psychiatrically complex populations.

## Data Availability

The original contributions presented in the study are included in the article/**Supplementary Material**. Further inquiries can be directed to the corresponding author.
