# Supplementary material for: Module-specific diagnostic accuracy of ADOS-2 in real-world clinical referral populations: an updated systematic review and HSROC meta-analysis
Source: Front Psychiatry. 2026 Jun 26;17:1840734. doi: 10.3389/fpsyt.2026.1840734 (PMC13350443; doi:10.3389/fpsyt.2026.1840734)
Supplement: Supplementary file 1 [file Table1.docx]

**Supplementary Table S1.** Study-Level 2×2 Diagnostic Accuracy Data (TP, FP, FN, TN) Used for HSROC Meta-Analysis.

| **Study** | **Module** | **True**  **Positive** | **False**  **Positive** | **False**  **Negative** | **True**  **Negative** | **Extraction Type** |
| --- | --- | --- | --- | --- | --- | --- |
| Feehan 2023 | M3–4 | 41 | 17 | 3 | 23 | Direct |
| Colombi 2020 | Module 3 | 27 | 10 | 8 | 13 | Derived (reported sens/spec) |
| Colombi 2020 | Module 4 | 19 | 9 | 4 | 13 | Derived |
| Nakamura 2024 | Module 4 | 37 | 21 | 13 | 19 | Derived |
| Greene 2021 | Module 3 | 55 | 34 | 1 | 124 | Derived |
| Gunderson 2025 | Toddler | 33 | 0 | 0 | 11 | Direct |

M3–4 = Combined Modules 3 and 4; HSROC = Hierarchical Summary Receive Operating Characteristic
